# Supplementary material for: A novel mechanosensitive channel controls osmoregulation, differentiation, and infectivity in Trypanosoma cruzi
Source: eLife. 2021 Jul 2;10:e67449. doi: 10.7554/eLife.67449 (PMC8282336; doi:10.7554/eLife.67449)
Supplement: Supplementary file 1. — Bold letters indicate restriction sites. Protospacer sequences are underlined, and ultramer sequences that correspond to the flanking regions of the gene are in italics. [file elife-67449-supp1.docx]

**Table 1. Sequences of primers used for genetic constructs and PCR screening.** Bold letters indicate restriction sites. protospacer sequences are underlined, ultramer sequences that correspond to the flanking regions of the gene are in italics

| Primer No. | Primer Name | Sequence |
| --- | --- | --- |
| 1 | BamHI-TcMscS Forward | 5’-**GGATCC**ATGAAACGCTTTTTCAATCGCT-3’ |
| 2 | HindIII-TcMscS Reverse | 5’- **AAGCTT**TCACTGCTTGGTTGCGTTGT-3’ |
| 3 | Blasticidin Forward | 5’-ATGGCCAAGCCTTTGTCTCA-3’ |
| 4 | Blasticidin Reverse | 5’-TTAGCCCTCCCACACATAAC-3’ |
| 5 | -167 bp TcMscS downstream | 5’TGGCACGATCGGTGTCGATA-3’ |
| 6 | -400bp TcMscS downstream | 5’**-** GTAATTTGTCCCTTCCTGGG-3’ |
| 7 | sgRNA 1 Forward BamHI | 5’‑GATC**GGATCC**GTAAGCGCTTTTCTGGTGCAGTTTTAGAGCTAGAAATAGC-3’ |
| 8 | sgRNA 2 Forward BamHI | 5’‑GATC**GGATCC**GGTGTAACAGGGGCCACAATGTTTTAGAGCTAGAAATAGC-3’ |
| 9 | sgRNA 3 Forward BamHI | 5‑GATC**GGATCC**GGTCAACAGTCAATTCGAACGTTTTAGAGCTAGAAATAGC-3’ |
| 10 | sgRNA Reverse | 5’‑CAGTGGATCCAAAAAAGCACCGACTCGGTG-3’ |
| 11 | 100 bp 3’-end-Blasticidin Forward Ultramer | 5’*AACATTTGTGATGAAACGCTTTTTCAATCGCTTCTATCTTGACACTGGCATTATTGCTGACCCCAGTCAACGTAGCCTCGCTAGTCGAGTAAGCG*C*TTTT*ATGGCCAAGCCTTTGTCTCA-3’ |
| 12 | 100 bp of 3’-UTR-Blasticidin Reverse Ultramer | 5’*TCACTGCTTGGTTGCGTTGTCTCCAGGTGTCTTGACAACGTCTTGTTTAGGGAGATCTGTTTCATGTGGGTTCTGCTTCTTTTCTTGTTCCCATGTCACT*TTAGCCCTCCCACACATAAC-3’ |
| 13 | 100 bp 5’-UTR-Blasticidin Forward Ultramer | 5’*CGCTGCGCCTTTGCTTGTTGGCGTGTCGAACACTCTTTTTTTTTTTTTTTTTTTTGCTTTTTTTCTTTAACGCGTCGGTGAAGAGAGAGAAACATTTGTG*ATGGCCAAGCCTTTGTCTCA-3’ |
| 14 | 3’-UTR-Blasticidin Reverse Ultramer | 5’*CGGAGTTCAGAAAACCCAGCGAGACATGGTGAAACACAAGACGTTCTCAAACACATTCTTATTCCTCATTCCTTACTCCAGAAAAGTAAAGGTCACTCCC*TTAGCCCTCCCACACATAAC-3’ |
| 15 | GAPDH Forward | 5’- CAGAGCCTCAGTGTTGGTG-3’ |
| 16 | GAPDH Reverse | 5’-TCAATACTCACTCTTGTTTGG-3’ |
| 17 | PAM-MutationForw | 5’-CAGGGGCCACAATTGCATTTGCTTGCAAGGA-3’ |
| 18 | PAM-MutationRev | 5’-TCCTTGCAAGCAAATGCAATTGTGGCCCCTG-3’ |
| 19 | HindIII-TcMscS Forw | 5’-**AAGCTT**ATGAAACGCTTTTTCAATCGCT-3’ |
| 20 | Xho-TcMscSnonstopRev | 5’-CTTAGG**CTCGAG**CTGCTTGGTTGCGTTGTC-3’ |
| 21 | HindIII-TbMscS Forw | 5’-CTTAGG**AAGCTT**ATGAAGCGATTTTTTGACAAG-3’ |
| 22 | Xho-TbMscSnonstopRev | 5’-CTTAGG**CTTGAG**CCTGACTTTACTTCGTTC-3’ |
