# Supplementary material for: A novel mechanosensitive channel controls osmoregulation, differentiation, and infectivity in Trypanosoma cruzi
Source: eLife. 2021 Jul 2;10:e67449. doi: 10.7554/eLife.67449 (PMC8282336; doi:10.7554/eLife.67449)
Supplement: Supplementary file 2. — Peak and recovery analysis of epimastigotes’ cell volume changes under hypoosmotic stress. For all the conditions, values are the mean ± SE of n = 6 independent experiments. p-values were calculated based on one-way analysis of variance with Bonferroni post-test. Differences were considered significant when p<0.01(*). [file elife-67449-supp2.docx]

**Table 2: Changes in cell volume upon hypoosmotic stress**

|  | **WT** | **Cas9** | **TcMscS-KD** | **TcMscS-KO** |
| --- | --- | --- | --- | --- |
| Peak (%) | 25.83±0.52 | 23.08±0.23 | 39.10±0.63* | 38.96±1.64* |
| p-value |  | 0.575 | 2.43E^-09^ | 8.65E^-05^ |
| Recovery slope | -0.0360±0.0029 | -0.0327±0.0057 | -0.0471±0.0031 | -0.0685±0.002* |
| p-value |  | 0.629 | 0.025 | 7.3E^-06^ |
| Final volume | 8.86±0.71 | 7.99±2.05 | 20.48±0.65* | 17.60±0.68* |
| p-value |  | 0.7099 | 2.96E^-07^ | 4.95E^-06^ |

For all the conditions values are Mean±SE of n=6- p values were calculated based on one-way ANOVA analysis with Bonferroni post-test. Differences were considered significant when p<0.01(*).
