# Supplementary material for: A novel mechanosensitive channel controls osmoregulation, differentiation, and infectivity in Trypanosoma cruzi
Source: eLife. 2021 Jul 2;10:e67449. doi: 10.7554/eLife.67449 (PMC8282336; doi:10.7554/eLife.67449)
Supplement: Supplementary file 3. — Peak and recovery analysis of epimastigotes’ cell volume changes under hyperosmotic stress. For all the conditions, values are the mean ± SE of n = 6 independent experiments. p-values were calculated based on one-way analysis of variance with Bonferroni post-test. Differences were considered significant when p<0.01(*). [file elife-67449-supp3.docx]

**Table 3: Changes in cell volume upon hyperosmotic stress**

|  | **WT** | **Cas9** | **TcMscS-KD** | **TcMscS-KO** |
| --- | --- | --- | --- | --- |
| Peak (%) | -12.41±1.36 | -15.78±0.74 | -22.43±1.12* | -20.10±1.59* |
| p-value |  | 0.0055 | 0.0002 | 0.0043 |
| Final volume | -14.38±0.93 | -11.59±1.08 | -23.84±0.71* | -23.56±0.82* |
| p-value |  | 0.087 | 4.27E^-05^ | 7.99E^-05^ |

For all the conditions values are Mean±SE of n=6. p values were calculated based on one-way ANOVA analysis with Bonferroni post-test. Differences were considered significant when p<0.01(*).
