# Supplementary material for: A novel mechanosensitive channel controls osmoregulation, differentiation, and infectivity in Trypanosoma cruzi
Source: eLife. 2021 Jul 2;10:e67449. doi: 10.7554/eLife.67449 (PMC8282336; doi:10.7554/eLife.67449)
Supplement: Supplementary file 4. — The number of intracellular amastigotes was counted at 6 and 48 hr post-infection. For all the conditions, values are the mean ± SE of n = 5 independent experiments. p-values were calculated based on one-way analysis of variance with Bonferroni post-test. Differences were considered significant when p<0.05(*). [file elife-67449-supp4.docx]

**Table 4: Quantification of intracellular amastigotes**

| **Amas/100 cells** | **WT** | **Cas9** | **TcMscS-KD** | **TcMscS-KO** |
| --- | --- | --- | --- | --- |
| Average 6 h-pi | 2.49±0.90 | 1.41±0.65 | 3.46±0.96 | 3.09±1.26 |
| p-value |  | 0.36 | 0.48 | 0.71 |
| Average 48 h-pi | 56.43±5.73 | 54.96±5.20 | 37.12±1.17* | 25.64±1.06* |
| p-value |  | 0.85 | 0.029 | 0.006 |

For all the conditions values are Mean±SE of n=5. p values were calculated based on one-way ANOVA analysis with Bonferroni post-test. Differences were considered significant when p<0.05(*).
