# Supplementary material for: A novel mechanosensitive channel controls osmoregulation, differentiation, and infectivity in Trypanosoma cruzi
Source: eLife. 2021 Jul 2;10:e67449. doi: 10.7554/eLife.67449 (PMC8282336; doi:10.7554/eLife.67449)
Supplement: Supplementary file 5. — Extracellular trypomastigotes collected from the supernatant of infected cells were counted at days 4, 5, and 6 post-infection. For all the conditions, values are the mean ± SE of n = 4 independent experiments. p-values were calculated based on one-way analysis of variance with Bonferroni post-test. Differences were considered significant when p<0.05(*). [file elife-67449-supp5.docx]

**Table 5: Quantification of extracellular trypomastigotes**

| **Trypomastigotes (10^6^)** | **WT** | **Cas9** | **TcMscS-KO** |
| --- | --- | --- | --- |
| 4 dpi | 2.11±0.74 | 4.04±1.09 | 0.41±0.03 |
| 5 dpi | 12.6±4.34 | 14.2±5.19 | 1.83±0.48 |
| 6 dpi | 31.8±6.91 | 49.3±11.2 | 5.22±1.27* |
| p-value (6 dpi) |  | 0.24 | 0.003 |

For all the conditions values are Mean±SE of n=4. p values were calculated based on one-way ANOVA analysis with Bonferroni post-test. Differences were considered significant when p<0.05(*).
